# Preparation and Biochemical Characterization of *Penicillium crustosum* Thom P22 Lipase Immobilization Using Adsorption, Encapsulation, and Adsorption–Encapsulation Approaches

**DOI:** 10.3390/molecules30030434

**Published:** 2025-01-21

**Authors:** Ismail Hasnaoui, Sondes Mechri, Ahlem Dab, Nour Eddine Bentouhami, Houssam Abouloifa, Reda Bellaouchi, Fawzi Allala, Ennouamane Saalaoui, Bassem Jaouadi, Alexandre Noiriel, Abdeslam Asehraou, Abdelkarim Abousalham

**Affiliations:** 1 Génie Enzymatique, Membranes Biomimétiques et Assemblages Supramoléculaires (GEMBAS), Institut de Chimie et de Biochimie Moléculaires et Supramoléculaires (ICBMS), UMR 5246 CNRS, Univ Lyon, Université Lyon 1, Bât Raulin, 43 Bd du 11 Novembre 1918, F-69622 Villeurbanne cedex, France; ismail.hasnaoui9@gmail.com (I.H.); dabahlaam@gmail.com (A.D.); alexandre.noiriel@univ-lyon1.fr (A.N.); 2 Laboratoire de Bioressources, Biotechnologie, Ethnopharmacologie et Santé (LBBES), Faculté des Sciences d’Oujda (FSO), Université Mohammed Premier (UMP), Bd Mohamed VI BP 717, Oujda 60000, Morocco; noureddine.bentouhami@ump.ac.ma (N.E.B.); r.bellaouchi@ump.ac.ma (R.B.); e.saalaoui@ump.ac.ma (E.S.); a.asehraou@ump.ac.ma (A.A.); 3 Laboratoire des Biotechnologies Microbiennes et Enzymatiques et de Biomolécules (LBMEB), Centre de Biotechnologie de Sfax (CBS), Université de Sfax (USF), Route de Sidi Mansour Km 6, BP 1177, Sfax 3018, Tunisia; sondes.mechri@yahoo.com (S.M.); bassem.jaouadi@cbs.rnrt.tn (B.J.); 4 Research Unit of Microbiology, Biomolecules and Biotechnology, Laboratory of Chemistry Physics and Biotechnology of Molecules and Materials (LCPBMM), Faculty of Sciences and Techniques Mohammedia (FSTM), Hassan II University of Casablanca, BP 146, Mohammedia 28806, Morocco; houssam.abouloifa@gmail.com; 5 Laboratoire de Biologie Cellulaire et Moléculaire (LCMB), Equipe de Microbiologie, Faculté des Sciences Biologiques (FSB), Université des Sciences et de la Technologie Houari Boumediene (USTHB), El Alia, Bab Ezzouar, Alger 16111, Algeria; fawzi.allala_fsb@usthb.edu.dz

**Keywords:** *Penicillium crustosum* lipase, immobilization, hybrid carriers, esterification

## Abstract

This work describes the immobilization and the characterization of purified *Penicillium crustosum* Thom P22 lipase (PCrL) using adsorption, encapsulation, and adsorption–encapsulation approaches. The maximum activity of the immobilized PCrL on CaCO_3_ microspheres and sodium alginate beads was shifted from 37 to 45 °C, compared with that of the free enzyme. When sodium alginate was coupled with zeolite or chitosan, the immobilization yield reached 100% and the immobilized PCrL showed improved stability over a wide temperature range, retaining all of its initial activity after a one-hour incubation at 60 °C. The immobilization of PCrL significantly improves its catalytic performance in organic solvents, its pH tolerance value, and its thermal stability. Interestingly, 95% and almost 50% of PCrL’s initial activity was retained after 6 and 12 cycles, respectively. The characteristics of all PCrL forms were analyzed by X-ray diffraction and scanning electron microscopy combined with energy dispersive spectroscopy. The maximum conversion efficiency of oleic acid and methanol to methyl esters (biodiesel), by PCrL immobilized on CaCO_3_, was 65% after a 12 h incubation at 40 °C, while free PCrL generated only 30% conversion, under the same conditions.

## 1. Introduction

Many methods of immobilizing enzymes have been developed since 1950, and the term “immobilized enzyme” was defined in 1970 [1]. The desirable properties of enzymes and their wide range of industrial applications are frequently hindered by their limited shelf life, lack of operational stability, labor-intensive recovery process, and reprocessing [2]. Generally, the stability of enzymes in their free (soluble) form is limited. Furthermore, being soluble, their reuse is difficult, which makes their application on an industrial scale limited and expensive [3]. Enzyme immobilization on a range of supports, including inorganic materials and inert polymers, is now a necessary procedure in many industrial applications. This approach promotes better stability at high pH values and temperatures [4]. The different enzyme immobilization techniques are based on chemical and physical principles. Lipases are carboxylester hyrolases that catalyze the hydrolysis of *sn*-1 and *sn*-3 ester linkages in triglycerides (TGs). These enzymes are also capable of catalyzing ester synthesis in a poorly hydrated organic medium through transesterification reactions involving an exchange of acyl chains between a TG and an alcohol (alcoholysis) or a fatty acid (acidolysis), and interesterification reactions involving an exchange of acyl chains between two TGs [5]. The search for useful lipases with distinct activities and specificity remains an ongoing challenge for various industrial applications, such as detergents and food processing [6]. Enzyme-based detergents are now dominating the market in developed countries, representing over half of all detergents [7]. Microbial lipases are endowed with interesting biochemical and functional properties, giving them the capacity to catalyze hydrolysis and synthesis reactions in various industrial fields [7,8,9]. Esterification is one of the synthetic reactions that can be catalyzed by microbial lipases [10]. However, the use of these enzymes in their soluble form has certain disadvantages, such as their high cost and their denaturation in reaction systems, which leads to lower conversion rates. To overcome these problems, immobilization is an effective way to render these enzymes more robust, stable, reusable for successive cycles, and, consequently, efficient on an industrial scale [4,11,12]. The most common method of immobilization involves trapping the enzyme in a polymer using physical and chemical approaches. Physical immobilization methods include entrapment (encapsulation) of enzymes in polymers, such as alginate and polyacrylamide, adsorption onto an organic or inorganic carrier, and micro-encapsulation where the enzyme is inside a spherical capsule [13]. In the latter case, the enzyme is attached by reversible low-energy bonds, which means that they can become detached during washing [14,15]. Adsorption is the simplest, fastest, and most profitable method which consists of the reversible fixation of the enzyme on a support. In this case, the enzymes are strongly attached or adhere to the surface of support through the combination of hydrophobic interactions. This technique generally involves incubating the enzyme solution on the support to allow time for adsorption to occur.

The screening of certain parameters, such as pH, temperature, contact time, agitation, enzyme concentration, and the specific surface area of the support, is essential to prevent desorption and denaturation phenomena. Subsequently, any unbound enzyme residues are removed by washing [16]. For immobilization by entrapment in a polymer matrix, the enzyme is trapped inside a polymer (gel or film), for example, agar, cellulose triacetate, chitosan (CS), polyacrylamide, calcium alginate, or collagen [17]. These supports have gelling power but without any toxic effects [18]. However, the difficult passage through the substrate network is the major disadvantage of this method, with the critical parameter being the choice of matrix pore size. If the pores are very large, there is a risk of protein leakage, but the space must be sufficient to ensure the circulation of the substrate and its transfer to the active site of the enzyme. Immobilization by inclusion improves the mechanical stability of the enzyme and, in some cases, its thermostability and tolerance to organic solvents [16,19]. Chemical immobilization consists of creating covalent bonds between the support and the functional groups of the enzyme. The covalent bond prevents dissociation of the enzyme and gives better stability. However, the enzyme can lose part of its catalytic activity through the interaction with the active site on the support [17,20]. Co-crosslinking is one of the chemical immobilization methods which consists of the formation of intermolecular covalent bonds between the enzyme and the matrix using bi-functional reagents, such as di-isocyanate and glutaraldehyde, or by creating an ionic bond between the enzyme and a charged matrix, thus binding the enzyme to the matrix by affinity [21].

This technique is very useful, but its disadvantage is that it requires an alkaline pH to improve the reactivity of the nucleophilic groups present on the surface of the proteins. Some enzymes, such as *Candida rugosa* lipase, lose part of their activity with this type of immobilization [22].

Most studies relating to a specific use of enzymes have been carried out either in a laboratory or, at best, in pilot tests under idealized conditions with numerous simplifications [4]. The industrial scale-up of the enzymatic approach still faces some obstacles. The screening of specific conditions for producing the enzymes in different strains, production costs of enzymes, their stability with respect to storage and application conditions, the presence of inhibitors, etc., are all constraints that slow down the emergence of this technology [23]. Overcoming these obstacles requires the production of robust catalysts with improved intrinsic properties. Enzyme immobilization is one of the strategies applied to achieve this as it improves the characteristics of the enzymes making them more stable under high temperatures, pH, organic solvents, inhibitors, and changes in environmental conditions. As a result, immobilized enzymes exhibit better stability and cost-effectiveness, compared to their counterparts, in free and soluble forms [4,11].

However, the stability of the immobilized enzyme depends on the intrinsic nature of the enzyme, the immobilization conditions, the nature of the support used, and the reaction conditions.

No immobilization research regarding lipase activity from the genus *Penicillium* has been found in the literature, and for the first time with the current study, research into the immobilization and biotechnological applicability of the lipase enzyme from strain *Penicillium crustosum* Thom strain P22 was investigated.

The objective of this research was, therefore, the immobilization of lipase from *Penicillium crustosum* Thom strain P22 (called PCrL), an extracellular serine alkaline lipase previously purified by the authors [24], using adsorption, encapsulation, and adsorption–encapsulation approaches. Moreover, the present study describes the possible applications of this PCrL immobilized form in esterification reactions in non-aqueous media.

## 2. Results

### 2.1. Immobilization of PCrL and the Characterization of Different Forms

**Figure 1:**
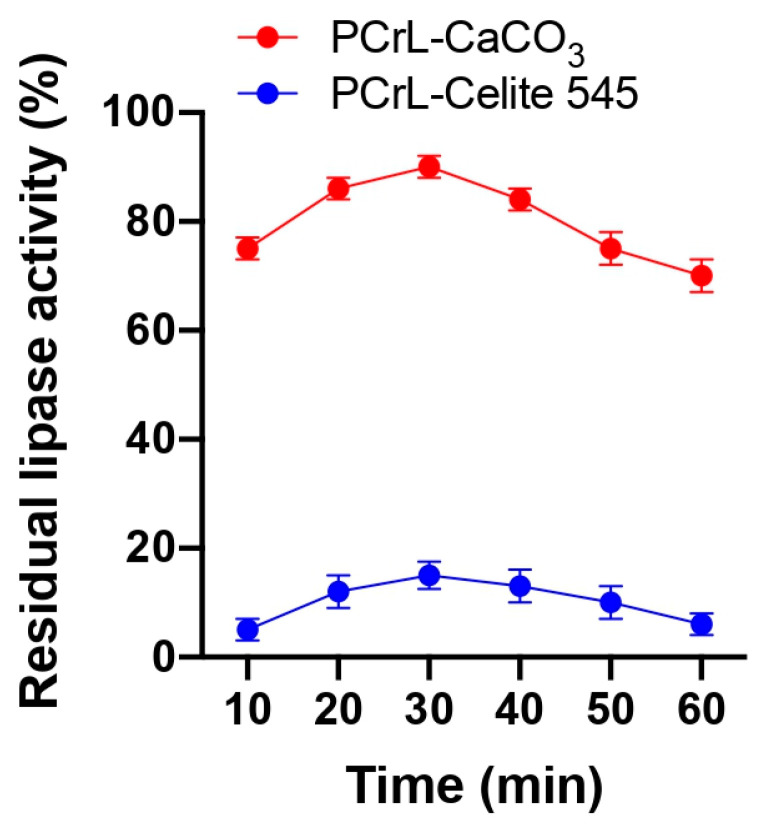
Adsorption kinetics of PCrL on CaCO_3_ and Celite 545. PCrL adsorbed on CaCO_3_ leads to the highest yield of 90% after 30 min of incubation (4500 U) at 4 °C. The lipase activity was measured with the pH-STAT technique using TC8 as the substrate.

The immobilization of PCrL by adsorption was carried out by incubating PCrL with CaCO_3_ or Celite 545. The yield percentages (Equations (1)–(3)) of the immobilized lipase activity obtained as a function of time are presented in Figure 1.

The results showed that CaCO_3_ leads to the highest yield of 90% after 30 min of incubation (4500 U) at 4 °C. On the other hand, PCrL adsorbed on Celite 545 showed a very low percentage of immobilization, up to a maximum of only 15%, after 30 min of incubation (750 U) at 4 °C (Figure 1). The strong binding of PCrL with CaCO_3_ could explain this increase in the immobilization of lipase activity. Indeed, this support is an adsorbent that leads to a widespread dispersion of PCrL on the support and preserves the catalytic activity. Therefore, CaCO_3_ was chosen as a suitable support for the immobilization of PCrL and it has the additional advantage of being non-toxic and devoid of any chemical reactivity. Two strategies were utilized to increase the effectiveness of PCrL immobilization: encapsulation with organic supports and encapsulation–adsorption with hybrid supports (organic–inorganic). For the immobilization of PCrL, we proceeded either with the use of SA alone, as an organic support, or with the use of SA combined with other supports, either inorganic (ZE) or organic (CS).

Conditions for the formation of SA gel beads were first optimized by varying several key parameters, such as SA and CaCl_2 _ concentrations, gelation time, pH, and temperature. We first varied the concentration of SA, from 0.5% to 4%, to assess the impact on gel bead formation. At lower concentrations, the beads were too fragile, leading to incomplete gelation and potential PCrL leakage. Higher concentrations (above 3%) resulted in beads that were too rigid, potentially hindering mass transfer and PCrL accessibility. We found that a concentration of 2% SA produced the most stable and functional beads, with sufficient rigidity to retain PCrL while maintaining a reasonable surface area for PCrL enzymatic activity. CaCl_2 _ was used as a cross-linking agent to stabilize the SA matrix. We tested a range of CaCl_2 _ concentrations, from 0.5 M to 3 M, to assess gelation efficiency. A concentration of 2 M CaCl_2 _ was found to provide the optimum cross-linking effect, resulting in homogeneous, spherical beads with a smooth surface and minimal leakage of the immobilized PCrL. Lower concentrations led to incomplete gelation, while higher concentrations caused bead aggregation, reducing the surface area and loading efficiency of PCrL. The gel bead size was controlled by adjusting the drop size during the gelation process. Using a Slip syringe, we varied the drop volume from 50 μL to 500 μL and assessed the impact on final bead size and enzyme activity. The smaller beads (50–100 μL) offered greater surface area relative to their volume but at the cost of greater fragility. The larger beads (300–500 μL) were more robust, but PCrL activity per bead was lower due to the reduced surface area available for interaction. After optimization, a bead size of 200 μL was selected as the compromise between mechanical stability and surface area.

We subsequently optimized the hardening time of the beads in the CaCl_2 _ solution, testing incubation times ranging from 15 min to 1 hour. Shorter times (15–30 min) resulted in weaker beads that easily disintegrated upon handling, while longer incubation times (over 45 min) did not significantly improve mechanical stability but led to a loss of PCrL activity. An optimal incubation time of 30 min was selected for bead stabilization. After optimizing gel bead formation, we tested different enzyme loading conditions by varying the concentration of lipase in the 2% SA solution. Higher concentrations of PCrL led to increased PCrL loading per bead, but also to a higher risk of leakage during washing. Based on these results, we selected a lipase concentration of 10 mg/mL for optimal PCrL loading without significant loss during handling.

As pH and temperature can affect SA solubility and cross-linking efficiency, we tested different pH values (from 4.5 to 7.5) and temperature (from 4 °C to 30 °C) conditions during bead formation. The gelation process was most efficient at a neutral pH value (around 7) and at room temperature (25 °C), allowing optimal retention of PCrL without affecting the structural integrity of the gel beads. These selected factors enabled us to form stable, homogeneous SA gel beads with high enzyme loading capacity, minimal leakage, and maximum PCrL activity after immobilization.

The CS and ZE used in this study are eco-friendly binders to alginate-PCrL and show great potential in the application of immobilized PCrL in various industries, such as detergents and food processing. We adjusted the concentrations of various supports, the incubation period, and the CaCl_2_ concentration in order to enhance the immobilization yield, the retained activity, and the minimal amount of PCrL release. Immobilization yields of 94 and 95% were observed using PCrL-SA and PCrL-SA-CS with recovery activities of 19.9 and 27.2%, respectively (Table 1). When SA was coupled with ZE as the support, the immobilization yield reached 100% with an improvement in activity recovery of about 47.3% (Table 1). Chemical modification of the immobilized enzyme can occasionally cause minor conformational changes in PCrL, which might reduce catalytic efficiency.

### 2.2. XRD Analysis of Immobilized PCrL

XRD is a widely used tool to acquire information on the structure of the crystal lattice and the degree of crystallinity in the microstructure of the support-immobilized enzyme.

This analysis is important because the structural integrity of the support can directly affect the stability of the enzyme. For instance, changes in diffraction patterns can indicate modifications in the crystal structure that may influence the thermal behavior of the immobilized enzyme.

**Figure 2:**
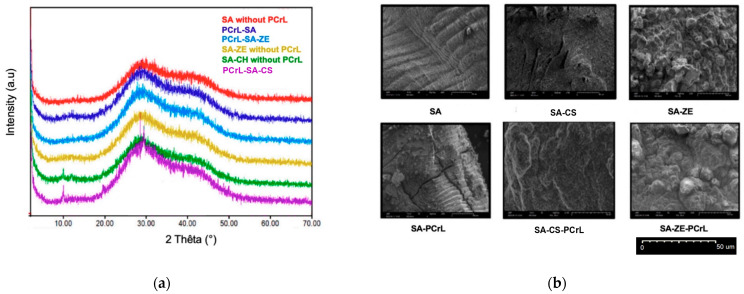
Identification of the PCrL-SA-ZE, PCrL-SA-CS, and PCrL-CaCO_3_ beads. (**a**) The XRD patterns of the support beads alone (SA, SA-ZE, or SA-CS) or complexed with PCrL (PCrL-SA, PCrL-SA-ZE, and PCrL-SA-CS). The XRD patterns were generated using Match! software (version 3.10.2.173); (**b**) Identification of the support beads (SA, SA-CS, and SA-ZE) and those complexed with PCrL (PCrL-SA, PCrL-SA-CS, and PCrL-SA-ZE) in the FE-SEM images.

As shown in Figure 2a, the XRD pattern of the support beads alone (SA, SA-ZE, or SA-CS) depicted a broad diffraction peak at 2θ = 28° and an additional peak at 2θ = 42°. XRD patterns of SA-CS beads before and after enzyme immobilization show no significant changes, indicating that the beads’ crystal structure remains intact. This is consistent with the fact that the enzyme is encapsulated in the beads rather than adsorbed on the surface. XRD patterns of the SA-ZE beads show distinct peaks corresponding to the zeolite, and slight peak shifts after enzyme immobilization. These shifts indicate interactions between the enzyme and the zeolite surface, consistent with adsorption. The overall integrity of the beads suggests that some of the enzyme is also encapsulated in the beads.

Based on the XRD results, all three support materials (CS, ZE, and SA) appear to be suitable for PCrL immobilization due to their rigid structures. Furthermore, we could infer that the immobilized PCrL could be easily detached using external permanent magnets, which is crucial for the subsequent use of the biocatalyst. Consequently, further studies, some of which are currently underway in our laboratories, to test the effectiveness of magnet-based detachment are required.

### 2.3. FE-SEM Analysis of Immobilized PCrL

The FE-SEM analysis allowed us to observe the surface morphology of the immobilized enzyme and the distribution of the PCrL on the support (Figure 2b). Both the support beads (SA, SA-CS, and SA-ZE) and those complexed with PCrL (PCrL-SA, PCrL-SA-CS, and PCrL-SA-ZE) exhibited a dense, abrasive, mesh-like surface structure in the micrographs (Figure 2b). A notable difference was observed in pore size. The pores were smaller in the immobilized PCrL beads compared to the support beads alone, which suggests that the enzyme molecules might be filling these pores (Figure 2b). The surfaces of SA-ZE beads displayed a rougher surface compared to SA beads alone, although this does not necessarily indicate enzyme leakage. For more details, the SEM images of SA-CS beads show a smooth, homogeneous surface, indicating that the enzyme is encapsulated in the gel matrix. The absence of surface irregularities or protrusions suggests that PCrL is uniformly distributed throughout the beads, consistent with encapsulation. The SEM images of the SA-ZE beads reveal surface roughness and the presence of lipase particles on the bead surface, indicating that the enzyme is adsorbed onto the surface. In addition, the beads display structural features that suggest that part of the PCrL is encapsulated inside, confirming the adsorption–encapsulation mechanism.

The SEM analysis allowed us to observe significant morphological changes in the CaCO_3_ beads after enzymatic treatment, suggesting potential enzyme incorporation. However, we acknowledge that these observations alone are not sufficient to confirm encapsulation or uniform distribution of PCrL. Annotations have been added to the SEM images to indicate structural features that may be associated with enzyme incorporation. However, we acknowledge that higher-resolution imaging or additional labeling techniques would be required for definitive confirmation.

SEM-EDS elemental mapping revealed the presence of nitrogen, a key marker for protein incorporation, on the bead surface. However, nitrogen distribution cannot definitively confirm enzyme encapsulation or its uniformity within the beads. These findings should be interpreted as complementary to morphological observations and PCrL activity assays.

Future studies could include SEM inspections before and after washing steps, enzyme staining, or post-complexation techniques combined with high-resolution EDS or transmission electron microscopy (TEM) analyses to provide more direct evidence of enzyme incorporation and distribution.

### 2.4. Biochemical Characterization of Immobilized PCrL

**Figure 3:**
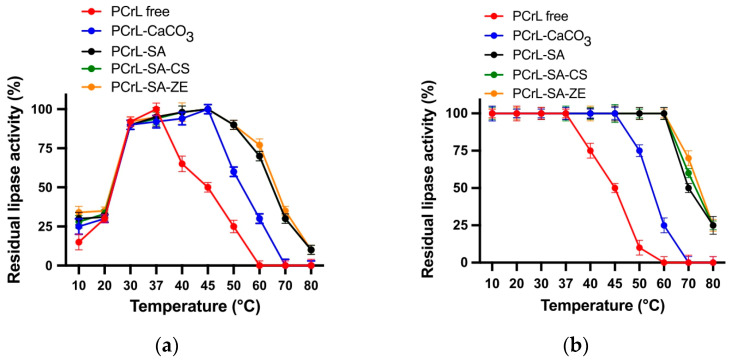
The effect of temperature on PCrL activity and stability. (**a**) The temperature–activity profile. This graph shows the activity of the free and immobilized PCrL at different temperatures. The activity of the immobilized PCrL at its optimal temperature (45 °C) is set at 100%. (**b**) Temperature stability. This graph illustrates the stability of the free and immobilized PCrL after incubation at different temperatures for 60 min. The residual activity was measured at pH value 9 using TC8 as a substrate. Each data point on the graph represents the average of three independent experiments.

**Figure 4:**
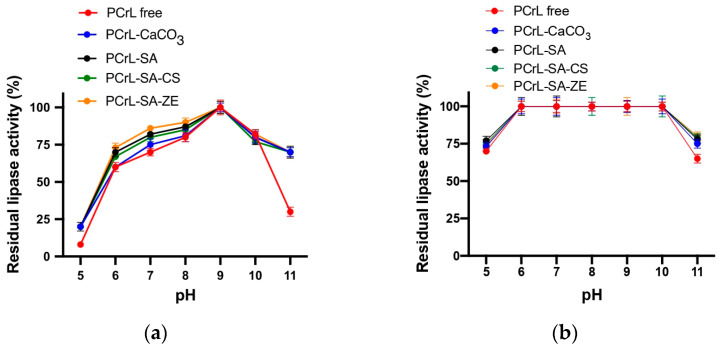
The effect of pH on PCrL activity and stability. (**a**) The pH activity profile of the free and immobilized PCrL at different pH values. The maximum activity at pH value 9 is set at 100%. (**b**) pH value stability of the free and immobilized PCrL after incubation at different pH values for 1 h at 4 °C. The residual activity was measured at pH value 9 and 37 °C using TC8 as the substrate. Each data point represents the average of three independent experiments.

The activity profiles of free and immobilized PCrL, at various temperatures, are shown in Figure 3a. As expected, and confirmed by our previous report [24], free PCrL exhibits peak activity at 37 °C. However, its activity significantly decreases at higher temperatures, retaining only 50% of its maximum activity at 45 °C (Figure 4a and [24]). In contrast, the immobilized PCrL (PCrL-SA, PCrL-SA-CS, and PCrL-SA-ZE) shows remarkable thermal stability. It maintains high activity across a broader temperature range (30–50 °C), with an optimal temperature of 45 °C (Figure 3a). Even at 50 and 60 °C, these immobilized forms retain a considerable portion of their maximum activity (86–92% and 65–77%, respectively) (Figure 3a). This is a significant improvement over PCrL-CaCO_3_, which shows a much steeper decline in activity at higher temperatures (Figure 3a).

Regarding thermostability, free PCrL is relatively unstable at elevated temperatures. After 60 min of incubation, it retains only 25%, 50%, and 10% of its initial activity at 40 °C, 45 °C, and 50 °C, respectively (Figure 3b). In contrast, immobilized PCrL (PCrL-SA, PCrL-SA-CS, and PCrL-SA-ZE) exhibits significantly enhanced thermal stability. It retains 100% of its activity after 60 min at 60 °C (Figure 3b). Even at 70 °C, these immobilized forms retain a substantial portion of their activity (50–70%) (Figure 3b). While PCrL-CaCO_3_ shows some improvement in thermal stability compared to the free enzyme, it is less stable than the SA-based immobilized forms. It retains only 25% of its activity after 60 min at 60 °C (Figure 3b).

Next, we investigated the impact of pH on the activity and stability of the immobilized PCrL (Figure 4). Immobilization did not alter the optimal pH for PCrL activity, which remained at 9 for both free and immobilized forms (Figure 4a). Interestingly, the immobilized PCrL exhibited significantly better activity at high pH values compared to the free enzyme. While free PCrL retained only 30% of its activity at pH value 11, the immobilized enzyme maintained 70% of its activity under the same conditions (Figure 4a).

Regarding the pH stability, both free and immobilized PCrL exhibit excellent stability across a wide pH range of 6–10 (Figure 4b). When incubated for 1 hour at 4 °C, both forms retain 100% of their initial activity within this pH range (Figure 4b).

### 2.5. Evaluation of the Performance of Immobilized PCrL in Organic Synthesis

**Figure 5:**
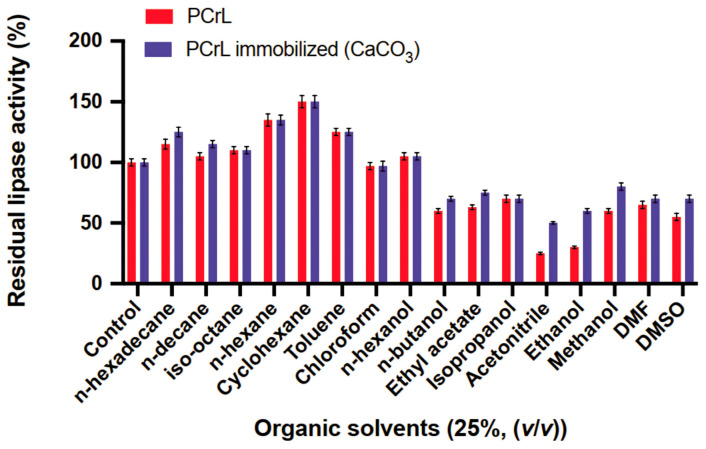
The effect of organic solvents on PCrL-CaCO_3_ activity and stability. The enzyme was incubated with 25% (*v*/*v*) of each solvent for 24 h. The residual activity was measured under standard conditions, using TC8 as the substrate at 37 °C and pH 9, as described in Material and Methods, and then expressed as a percentage of the activity without any solvents. Each data point represents the average of three independent experiments, with the error bars indicating standard deviation.

We investigated the impact of different organic solvents on the activity of both free and immobilized PCrL (Figure 5). After 24 h of incubation in 25% (*v*/*v*) water-immiscible organic solvents (Log *p* > 2.0), such as n-hexadecane, n-decane, iso-octane, n-hexane, cyclohexane, or toluene, the immobilized PCrL showed comparable stability to the free enzyme. In some cases, the immobilized enzyme even exhibited higher activity than the free enzyme (Figure 5).

Even with chloroform and n-hexanol, activity remained above 95% (Figure 5). These findings suggest that despite immobilization PCrL activity can be maintained, even in the presence of organic solvents commonly used for biocatalysis. However, the real advantage of immobilization becomes evident when using water-miscible organic solvents (Log *p* < 2.0), such as n-butanol, ethyl acetate, isopropanol, acetonitrile, ethanol, methanol, DMF, or DMSO (Figure 5). While free PCrL exhibited poor stability in these solvents, retaining only 25–70% of its activity, immobilized PCrL showed significantly higher stability, maintaining 50–80% of its activity (Figure 5). This enhanced stability in polar solvents is thought to be due to the protective effect of the support matrix, which shields the enzyme from denaturation.

**Figure 6:**
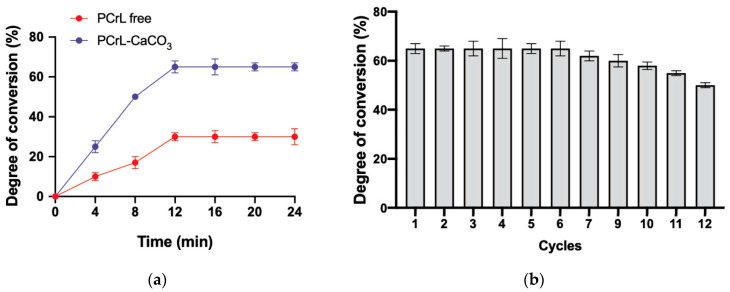
Performance evaluation of PCrL-CaCO_3_. (**a**) The kinetics of oleic acid esterification catalyzed by the free PCrL and immobilized PCrL-CaCO_3_. The reaction was performed at 40 °C, with stirring for 24 h, using 500 U of enzyme in hexane with a 3:1 molar ratio of methanol to oleic acid. (**b**) The reusability of immobilized PCrL-CaCO_3_ in multiple reaction cycles. The enzyme was reused for 12 cycles, with each cycle lasting 12 h. The conversion yield of oleic acid to esters was monitored for each cycle.

PCrL immobilized on CaCO_3_ exhibited exceptional stability in various organic solvents, maintaining over 50% activity after 24 h of incubation at a 25% (*v*/*v*) concentration. This stability is crucial for efficient biocatalysis. The immobilized PCrL demonstrated superior catalytic activity in the esterification of oleic acid to produce biodiesel. After 12 h of reaction at 40 °C, the immobilized enzyme achieved a 65% conversion yield, significantly outperforming free PCrL (30% conversion) (Figure 6a).

One of the major advantages of immobilized enzymes is their reusability. The immobilized PCrL maintained a 100% level of activity for the first six cycles, consistently yielding a 65% conversion rate (Figure 6b). A slight decrease in activity was observed after 12 cycles, possibly due to enzyme desorption or denaturation (Figure 6b).

### 2.6. Thermodynamic Parameters of the Thermal Inactivation of PCrL

Table 2 presents the kinetic and thermodynamic parameters of free and immobilized PCrL. The immobilized PCrL exhibited a longer half-life (t_1/2_) compared to the free enzyme, indicating increased stability. The decimal reduction time (D) also increased for the immobilized PCrL, further confirming its enhanced stability (Table 2). The higher activation energy (Ea) (Table 2) could be attributed to the increased distance between the enzyme and the substrate due to immobilization which may slightly hinder the formation of the enzyme-substrate complex.

The structural integrity observed by XRD (see Figure 2a) and the morphology seen by SEM (see Figure 2b) could give us indirect clues to the enzyme’s thermal behavior. For instance, a more ordered crystal structure and uniform distribution of the enzyme might enhance the thermal stability of the immobilized PCrL, whereas structural defects or uneven distribution of the enzyme could lead to thermal instability. Future studies should incorporate thermal analyses, such as differential scanning calorimetry or thermogravimetric analysis, to directly evaluate the thermal stability of immobilized PCrL. These techniques would complement structural and morphological characterizations and provide a more complete understanding of the enzyme’s stability under different thermal conditions.

**Figure 7:**
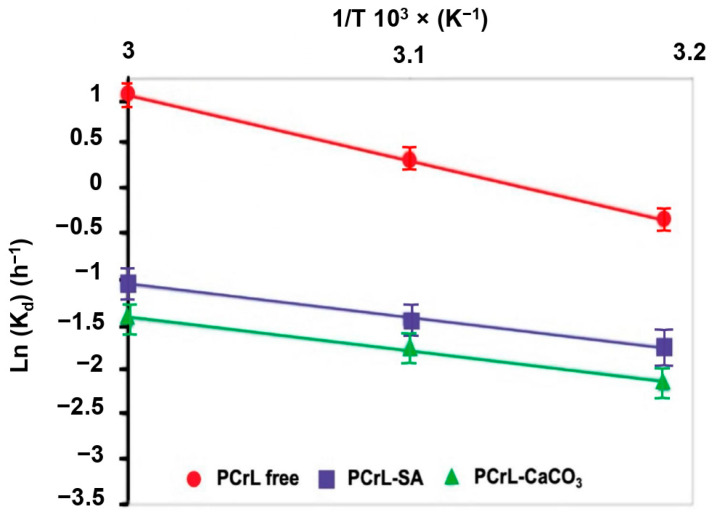
The thermodynamic parameters of the target PCrL. An Arrhenius diagram of Ln (*k*_d_) vs. 1/temperature to calculate the activation energy (E_a_).

Table 2 and Figure 7 explore the thermodynamic parameters affecting the activity of free and immobilized PCrL. These parameters reveal how temperature influences enzyme–substrate interactions and stability. The activation energy (Ea) required for the formation of the enzyme–substrate complex decreased upon immobilization (PCrL-SA and PCrL-CaCO_3_) (Figure 7). This suggests that immobilization facilitates the interaction between PCrL and the substrate molecule. The values of ΔH_d_ were generally higher for the immobilized PCrL compared to the free enzyme (Table 2). While ΔH_d_ slightly decreased with increasing temperature for all forms, the higher overall ΔH_d_ values for the immobilized PCrL indicate its improved thermal stability, i.e., more energy is required to disrupt or denature the structure of the immobilized enzyme at high temperatures. The ΔG_d_ values were also slightly higher for the immobilized PCrL compared to the free enzyme (Table 2). Positive ΔG_d_ values indicate that the non-denatured state of the enzyme is more favorable under these conditions. The increase in ΔG_d_ with immobilization suggests that the three-dimensional structure of the enzyme becomes more rigid and resistant to unfolding at elevated temperatures.

## 3. Discussion

A variety of complex organic and hybrid (organic–inorganic) carriers have been used as powerful supports for the immobilization of PCrL. In fact, we used supports like CaCO_3_, Celite 545, SA, CS, and ZE for the immobilization of the PCrL enzyme. Specifically, the combination of SA with ZE and CH as organic and inorganic supports, respectively, may be an innovative aspect. The study of immobilization efficiency and activity recovery in these various support configurations could distinguish our approach from other studies. The adsorption and encapsulation of the PCrL together is an innovative method that could offer benefits in terms of stability and catalytic efficiency. This dual approach to immobilization may not have been common before [25,26].

Enzyme activity assays provided functional evidence of PCrL immobilization, demonstrating that catalytic function was retained after incorporation. While this does not confirm the exact localization of PCrL within the beads, it supports the conclusion that immobilization was successful.

We have demonstrated in this work that immobilization significantly enhanced PCrL’s thermal, pH, and organic solvent stability, making it a promising candidate for industrial applications. The high immobilization yield of PCrL on CaCO_3_ aligns with previous studies. After a 30-min incubation of the enzyme with CaCO_3_, Ghamgui et al. [25] reported a 95% yield for *Rhizopus oryzae* lipase, while Ben Bacha et al. [26] achieved yields greater than 89% for *Bacillus stearothermophilus* and *Proteus vulgaris* OR34 lipases. These observations can be explained by the fact that CaCO_3_ consists of small crystalline particles that create a large surface area, facilitating efficient enzyme adsorption [27,28]. In addition, the use of CaCO_3_ as an immobilization matrix is based on its low cost, its non-toxic nature, and its lack of chemical reactivity with proteins, which minimizes enzyme denaturation [6,28]. It has been reported that immobilization by physical adsorption is a straightforward technique that relies on weak interactions (hydrophobic interactions, hydrogen bonds, and van der Waals forces) between the enzyme and the support, preserving enzyme activity [29]. The immobilization of *Yarrowia lipolytica* lipase, via adsorption on octyl-agarose and octadecyl-sepabeads, led to a high yield and excellent stability of the enzyme [30]. Furthermore, studies performed on *Candida rugosa* lipase, immobilized by adsorption on poly(3-hydroxybutyrate-co-hydroxyvalerate) or hydroxyapatite, showed conservation of activity above 90%, after 3 h at 60 °C or 4 h at 50 °C, respectively, with an enzyme reuse of 12 cycles [31]. While the weak nature of these interactions can lead to some enzyme desorption under certain conditions, it can be beneficial for applications in organic solvents. Lipases, which are poorly soluble in organic solvents, remain bound to the support, ensuring their continued catalytic activity [32].

Immobilization can sometimes hinder the active site of the enzyme, thereby reducing its accessibility to substrates, and chemical modifications during immobilization can induce minor conformational changes in the enzyme structure, which affect its catalytic efficiency [33]. To address these potential challenges, we employed the adsorption–encapsulation strategy, with hybrid supports, for the immobilization of PCrL. The combination of SA with ZE or CS offers several advantages, including enhanced enzyme adsorption by improving enzyme binding and minimizing leakage, together with the provision of excellent mechanical support that prevents enzyme detachment. Furthermore, both CS and ZE are environmentally friendly materials, making the process more sustainable.

The XDR results for the immobilized PCrL concur with previous findings [15]. In that earlier study, rigid structures, such as sodium alginate, white clay, and kaolin, were successfully used as supports for protease immobilization. The immobilized protease (SPSM) could be easily separated using magnets, thus enhancing its reusability. The thoroughness of our methodology, including structural characterization of PCrL beads by XRD and FE-SEM, as well as precise measurements of PCrL activity under various experimental conditions, distinguishes our work for its level of detail. Other studies may not cover all these aspects with the same precision [34,35,36,37].

Immobilization significantly improved the thermal stability of PCrL, allowing it to function at higher temperatures than the free enzyme. The increased activity of immobilized PCrL at these higher temperatures is probably due to diffusional factors. Elevated temperatures can enhance the accessibility of the substrate to the enzyme within the support matrix [16]. In addition, immobilization may limit the flexibility of the enzyme, thereby preventing its denaturation at high temperatures [28,38]. Immobilization also improved PCrL’s stability at higher pH values. This remarkable stability could be attributed to the maintenance of the enzyme’s 3D structure, even under extreme pH conditions [27,29]. Our findings align with previous research on immobilized lipases [1,25,39,40], which consistently demonstrates that immobilization can enhance thermal stability.

Immobilized lipases are valuable biocatalysts for synthetic reactions, such as biodiesel production, often carried out in organic solvents. These solvents are regularly used in enzymatic reactions to improve substrate solubility, reduce viscosity, and accelerate reactions [41]. However, it is crucial to select solvents that minimize enzyme inactivation. The stability of immobilized PCrL in organic solvents and the study of its reusability over multiple cycles of catalysis is a distinctive feature of our work. This focus on long-term reusability could set our research apart from other studies that may not emphasize this aspect [34,35]. Immobilized PCrL has shown exceptional stability in water-immiscible organic solvents, such as n-hexane. In general, it has been found that biodiesel production rates generally rise in accordance with an increase in the hydrophobicity (immiscibility with water) of the solvent used [28]. Conversely, hydrophilic (water-miscible) solvents tend to remove water molecules bound to the enzyme surface. This dehydration process, called denaturation, reduces enzyme activity and lowers conversion rates [42]. It has been reported that solvents with a Log *p* of less than 2 are more likely to remove the water of hydration from the enzyme, leading to deactivation, while solvents with a Log *p* between 2 and 4 are less likely to cause dehydration, providing a safer range for enzyme activity [43]. This explains the common use of n-hexane (Log *p* = 3.5) in biodiesel production. Its hydrophobicity promotes efficient reactions while remaining within a Log *p* range that is more favorable to enzymes [44].

The use of PCrL immobilized on CaCO_3_ for the synthesis of methyl esters, in the presence of hexane, has proven to be more efficient than the free enzyme. *Penicillium expansum* Lipase was also used to catalyze the transesterification reaction of corn oil with methanol, with a degree of conversion into biodiesel of 86% [45]. The lipases from *Rhizomucor miehei* and *Candida antarctica* in the transesterification of various oils, using hexane as a solvent, showed that lipase from *Rhizomucor miehei* is more effective in converting primary alcohols (methanol, ethanol, propanol, and 1-butanol), with yields of between 95 and 98%, while *Candida antarctica* lipase is more suitable for the conversion of secondary alcohols (isopropanol and 2-butanol), with yields of between 61 and 84% [45]. In addition, *Geotrichum candidum* lipase shows a conversion yield of 37% [46]. Lipase from *Proteus* sp. W1, expressed in *E. coli*, has also been used to catalyze a transesterification reaction of palm oil with ethanol. The degree of conversion into biodiesel was 43% [47]. Furthermore, the transesterification of rapeseed oil with methanol, catalyzed by lipase from *Proteus* sp. K107 (expressed in *E. coli*), showed a conversion efficiency of 97% [48].

A significant advantage of immobilized enzymes is their reusability. The immobilized PCrL retained almost 100% of its initial activity after 6 cycles and was still found to be at half of its original activity after 12 cycles. This reduction could be explained by either the enzyme partially desorbing from the support or partially denaturing as a result of prolonged exposure to the reaction medium. However, it has been demonstrated by other authors that immobilized lipases can catalyze subsequent reactions with the same yield after multiple cycles [49,50]. This analysis highlights the potential of immobilized PCrL for various industrial applications, particularly in the field of biocatalysis where its improved stability, activity, and reusability make it a valuable biocatalyst.

In addition, PCrL immobilization significantly reduces enzyme consumption in industrial processes. By reusing the immobilized enzyme over several cycles, the need for constant replenishment of fresh enzymes is minimized, leading to a reduction in overall waste. This is particularly beneficial for industries where enzymes can be expensive and waste reduction is a priority. The supports used in the immobilization process, such as CaCO_3_, Celite 545, SA, CS, and ZE, are derived from natural, non-toxic materials. These materials are biodegradable or recyclable, in line with environmentally friendly practices. Furthermore, using these supports could reduce the need for synthetic chemicals that could harm the environment. The PCrL immobilization process and subsequent catalytic reactions, such as biodiesel production, can potentially be carried out under milder conditions (e.g., lower temperatures or ambient pH) than other conventional chemical synthesis methods, thus reducing energy consumption. In the case of biodiesel production, for instance, the immobilized PCrL could operate efficiently under less harsh conditions, lowering the energy and carbon footprint of the process. Immobilized PCrL can catalyze reactions in non-aqueous media, reducing dependence on hazardous chemicals and solvents commonly used in organic synthesis. This can reduce the environmental impact associated with solvent disposal and contamination, contributing to safer industrial processes.

In our work, the specific use of immobilized PCrL in the production of biodiesel through the esterification of oleic acid is a practical application of PCrL immobilization, which may differentiate our study from others that focus more on general enzyme immobilization techniques rather than targeted applications [34,35].

The use of first-order kinetic models to analyze the thermal inactivation of PCrL, as well as Arrhenius equations and thermodynamic calculations to estimate the activation energy and stability of PCrL under different conditions, appears to be a comprehensive approach that has perhaps not been as widely covered previously [34,35,36,37].

The increase in ΔG_d_ following immobilization suggests that the three-dimensional structure of the enzyme becomes more rigid and resistant to unfolding at elevated temperatures. The higher activation energy observed for the immobilized PCrL can be attributed to the increased distance between the enzyme and the substrate which could slightly hinder the formation of the enzyme–substrate complex. The results also suggest that immobilization improves the thermal stability of PCrL. While the increased activation energy might slightly reduce catalytic activity, the benefits of enhanced stability and reusability often outweigh this drawback. Overall, the thermodynamic data support the notion that immobilization enhances PCrL stability, particularly at higher temperatures. The modification of the 3D structure of PCrL is also complemented by an increase in the disturbance, randomness, or activation entropy, which agrees with the results of the activity and stability assays.

## 4. Material and Methods

### 4.1. Material

Glyceryl trioctanoate (TC8), bovine serum albumin (BSA), sodium carbonate (CaCO_3_), Celite 545, sodium alginate (SA), chitosan (CS), and zeolite (ZE) were obtained from Sigma-Aldrich Chimie (Saint-Quentin-Fallavier, France). PCrL was purified to homogeneity and biochemically characterized, as described previously [24]. The size of the CaCO_3_ and Celite particles used in our study is approximately 50 µm. While the particle size of Celite is around 20 µm.

### 4.2. Lipase Activity Measurements and Protein Determination

The lipase activity was measured potentiometrically, using a pH-STAT device (Metrohm 902 Titrando, Herisau, Switzerland) based on the automatic and continuous titration of fatty acids released by the action of lipase on a mechanically stirred TC8 emulsion used as the substrate. The titration was carried out with a 100 mM NaOH solution to maintain the reaction medium at a constant predetermined pH. Each enzymatic assay was performed in a thermostatically controlled container (37 °C) containing 0.5 mL of TC8, 14.5 mL of 0.3 mM Tris-HCl buffer (pH 9), 150 mM NaCl, and 2 mM CaCl_2_ [24]. Enzymatic activity was expressed in international units (IU) (1 IU corresponding to 1 μmol of fatty acids released per min). The protein concentration was determined according to the method of Bradford [51], using a reagent kit from Bio-Rad Dye, Hercules, CA, USA, and BSA as the standard.

### 4.3. PCrL Immobilization

The PCrL was immobilized by adsorption on CaCO_3_ (PCrL-CaCO_3_) or Celite 545 (PCrL-Celite 545), according to the protocol described previously [26]. Firstly, a volume of 1 mL of PCrL (0.1 mg/mL, 5000 U/mg, and 10,000 U/mg of olive oil and TC8, respectively) was brought into contact with 1 g of CaCO_3_ or Celite 545, with stirring, at 4 °C. The incubation time was enhanced by performing four immobilization tests, and each was incubated for a specific duration (0.5, 1, 2, or 3 h). Then, the mixture was filtered through Whatman paper and washed twice with 10 mL of cold acetone (−20 °C). Finally, the immobilized PCrL was dried in a vacuum desiccator at room temperature (25 °C) for 6 h and stored at 4 °C until use.

For the encapsulation method, SA was either used alone or combined with CS as an organic support. For the adsorption–encapsulation method, SA was used combined with ZE as an inorganic support. The SA was dissolved in hot sterilized deionized water passed through a 0.45 µm filter. The mixture obtained was placed for 1 h at 4 °C for cooling and degassing. The PCrL (500 U, 300 µL) was added to 700 µL of the appropriate buffer (0.3 mM Tris-HCl (pH 9) containing 150 mM NaCl and 2 mM CaCl_2_). Subsequently, SA (3 mL at 2%, *w*/*v*) was added to the PCrL solution (PCrL-SA). When SA was used, combined with another support, PCrL (500 U, 300 µL) was incubated with 700 µL of zeolite (PCrL-SA-ZE) or chitosan (PCrL-SA-CS) at concentrations of 1.2% *w*/*v* or 0.8% *w*/*v*, respectively, for 2 h at 4 °C, then added to the SA solution (3 mL). In both cases, the mixtures obtained were homogenized for 1 h in the cold with gentle stirring.

After homogenization, the PCrL-support mixture beads were added dropwise using a 10 mL syringe to a 200 mM CaCl_2_ solution. The bead formation was performed using 30 mL Luer Slip syringes, which provided a controlled and reproducible droplet size of 2 mm in diameter. The beads were immediately gelled by the crosslinking between the Ca^2+^ and SA. The resulting beads were then washed three times with deionized water, air-dried on Whatman paper, and weighed. Finally, the dried beads were stored in a suitable buffer at 4 °C for future use.

The yield of immobilized lipase activity was defined as the ratio of the immobilized activity (expressed in the total unit, U) recovered at the end of the immobilization period divided by the total soluble PCrL activity, initially added at 1 g of support (Equation (1)). The immobilized activity was determined by measuring the total residual enzyme activity that remains in the enzyme solution after immobilization (Unbound enzyme activity) and subtracting this activity from the initial total activity (Initial enzyme activity), as follows:

(1) $$Yield\;\left(\%\right)=\frac{\left(Initial\;enzyme\;activity-Unbound\;enzyme\;activity)(U\right)}{Initial\;enzyme\;activity\left(U\right)}\times 100$$

The immobilization efficiency refers to the activity of the PCrL observed in the support after immobilization (Equation (2)). It describes the percentage of immobilized lipase activity in the support, as follows:

(2) $$Efficiency\;\left(\%\right)=\frac{Observed\;activity\;in\;the\;support\;(U)}{\left(Initial\;enzyme\;activity-Unbound\;enzyme\;activity)(U\right)}\times 100$$

The PCrL recovery activity retained in the beads defines the percentage activity of the immobilized enzyme compared with the starting total activity (Equation (3)), as follows:

(3) $$Recovery\;activity\;\left(\%\right)=\frac{Immobiliz\;edenzyme\;activity\;(U)}{Initial\;enzyme\;activity\left(U\right)}\times 100$$

### 4.4. Characterization of PCrL Beads

Structural analyses of the PCrL beads were studied by X-ray diffraction (XRD) using an XRD-6000 (SHIMADZU, Kyoto, Japan) in the range of 2θ, an angle of 10–65° using Cu Kα radiation (λ = 0.154 nm), and a scanning speed of 4°/s [15]. The XRD models were generated using Match! software (version 3.10.2.173). Morphological analyses of the PCrL beads were carried out by emission scanning electron microscopy (FE-SEM) using a JEOL-JSM7001F apparatus (Tokyo, Japan). The working distance was achieved at 6 mm (the distance between the sample and the objective lens) and the SEM filament was run at variable currents, following a voltage of 5 kV when using various magnifications.

### 4.5. Biochemical Characterization of Immobilized PCrL

The optimal temperature for the activity of the immobilized PCrL was determined at pH value 9 and in a temperature range varying from 10 to 80 °C, using TC8 as the substrate. The relative activities (%) were calculated by comparison with the maximum activity obtained (100%). Thermal stability tests were performed by incubating the immobilized PCrL for 1 h at temperatures ranging from 20 to 80 °C, and the residual PCrL activity was measured under standard test conditions (pH 9 and 37 °C).

The activity of the immobilized PCrL was tested across a range of pH values (5 to 11) at 37 °C, using TC8 as the substrate. The pH level resulting in the highest activity (100%) was considered the optimum. To assess the immobilized PCrL’s pH stability, the immobilized enzyme was incubated for 1 h at 4 °C in various 50 mM buffers (pH 5–11). The residual activity was then measured under standard conditions (pH 9 and 37 °C).

The first-order model (Equation (4)) was used to determine the thermal inactivation of the PCrL:

(4) $$\ln\left[S\right]={-k}_{d}t+\ln{\left[S\right]}_{0}$$

where $\left[S\right]$ is the concentration of the substrate at time t, ${\left[S\right]}_{0}$ is the initial concentration of the substrate, and $k_{d}$ (min^−1^) is the deactivation first-order rate constant.

The half-time (t_1/2_) (Equation (5)) was defined as the time corresponding to a 50% reduction in activity:

(5) $$t_{1/2}=\frac{ln2}{k_{d}}$$

The time (D) required for a 90% reduction in activity was calculated as follows (Equation (6)):

(6) $$D=\frac{ln10}{k_{d}}$$

The deactivation rate constant ($k_{d}$) is derived from the Arrhenius equation (Equation (7)):

(7) $$\ln k_{d}=\ln A-\;\frac{E_{a}}{R}\times \frac{1}{T}$$

where A is the Arrhenius constant (min^−1^), $E_{a}$ the activation energy (kJ mol^−1^), R the universal gas constant (8.314 J mol^−1^ K^−1^), and T the absolute temperature (K).

The Gibbs free energy (${\Delta G}_{i}$, kJ mol^−1^), the enthalpy (${\Delta H}_{i}$, kJ mol^−1^), and the entropy (${\Delta S}_{i}$, J mol^−1^K^−1^) of the inactivation of PCrL were estimated according to the following equations (Equations (8)–(10)):

(8) $${\Delta G}_{i}=-\mathrm{RT ln}\frac{k_{d}\times h}{k_{b}\times T}\;$$

(9) $${\Delta H}_{i}=E_{a}-RT\;$$

(10) $${\Delta S}_{i}=\frac{{\Delta H}_{i}-{\Delta G}_{i}}{T}\;$$

where $k_{b}$ and *h* are the Planck constant (6.626 × 10^−34^ J s) and the Boltzmann constant (1.38 × 10^−23^ J K^−1^), respectively. To understand how temperature affects the stability of the immobilized enzyme, we used the rearranged absolute Eyring rate equation [15], derived from transition state theory, as follows (Equation (11)):

(11) $$k_{d}=\frac{k_{b}\times T}{h}\times e^{\frac{{\Delta H}_{i}}{RT}}\times e^{\frac{{\Delta S}_{i}}{R}}$$

### 4.6. Performance of Immobilized PCrL in Organic Synthesis

The organic solvent stability of the immobilized PCrL (CaCO_3_ and SA), compared to the free form of the enzyme, was studied by incubating the enzyme preparation with various organic solvents that have different partition coefficients (Log *p*), at 25% (*v*/*v*) for 24 h and at 37 °C with constant stirring at 200 rpm [27]. Aliquots were taken at the required time intervals to test for any remaining activity. The reaction mixture without any additive was taken as a control (100%).

The esterification of fatty acids with methanol, catalyzed by lipase, is a promising process for the production of biodiesel. Hence, we performed a PCrL-catalyzed esterification of oleic acid by methanol in non-aqueous media. The reaction was carried out in an airtight glass bottle at 37 °C, with stirring at 150 rpm, for 24 h. The reaction mixture was composed of 500 U of the immobilized PCrL, 6 mL of hexane, and a molar ratio of 3:1 methanol/oleic acid. A control with the same composition, but without the enzyme, was carried out under identical experimental conditions. Aliquots were taken every 4 h to determine the conversion rate of the reaction. For this, the immobilized enzyme was first recovered by centrifugation at 13,000× *g* for 5 min at 4 °C, then the residual fatty acids in the supernatant (in the oil phase) were solubilized in ethanol, followed by titration with 100 mM KOH, using phenolphthalein as a color indicator. The conversion rate was calculated as follows (Equation (12)):

(12) $$\mathrm{Degree of conversion}\;(\%)=\frac{A_{1}-A_{2}}{A_{1}}$$

where $A_{1}$ is the acid value of the control and $A_{2}$ is the acid value after esterification.

To test the reusability of the immobilized PCrL, the immobilized lipase was recovered by centrifugation, at 13,000× *g* for 5 min, after the esterification reaction (12 h), and then washed with hexane to eliminate any substrate or product retained in the support. Next, it was dried, under vacuum, and reused for another cycle of esterification using a new reaction mixture. A total of 12 successive cycles of esterification, using the same starting PCrL, were performed and the conversion degrees were calculated based on the acid value, as previously described.

All experiments were repeated at least three times (independent replicates). In addition, a control experiment without lipase was conducted under identical conditions. The results are presented as the average (mean) of these replicates, along with the standard deviation (±SD).

## 5. Conclusions

The immobilization of PCrL on different supports considerably improves its stability and reusability. The enzyme retained a high degree of activity over several reaction cycles, demonstrating its potential for long-term use in industrial applications. The immobilized PCrL was successfully used to catalyze the esterification of fatty acids with methanol in non-aqueous media, showcasing its potential for biodiesel production. This demonstrates the enzyme’s ability to operate efficiently under mild conditions, with reduced energy consumption compared to traditional chemical catalysts. The performance of the immobilized enzyme in organic synthesis suggests that it could replace traditional catalysts in a variety of other industrial processes, offering advantages in terms of specificity, reaction rate, and environmental sustainability. The immobilized PCrL exhibited remarkable thermal stability and pH tolerance, making it suitable for processes requiring varied conditions. This stability is a key factor for its industrial application in processes where conventional enzymes can be deactivated or lose activity over time. The immobilization process not only improves enzyme durability but also reduces operating costs by minimizing enzyme consumption and enabling reuse over several cycles. The environmental benefits include reduced waste production and less reliance on harmful chemicals. Future research could focus on further optimizing immobilization conditions (e.g., enzyme concentration, immobilization time, and support material) to maximize enzyme loading capacity and efficiency. The exploration of alternative supports or hybrid methods (such as combining natural polymers and inorganic supports) can improve the immobilization process, further enhancing enzyme stability and performance. Future studies should explore techniques to enhance the reusability of immobilized PCrL by improving its operational life through cross-linking or surface modifications to protect the enzyme from degradation during prolonged use. The potential for multi-cycle applications in large-scale industrial environments could further contribute to the technology’s economic viability. Developing processes that integrate immobilized PCrL into green chemistry frameworks could make it an integral part of sustainable biotechnology production. For instance, coupling immobilized lipase with renewable raw materials and non-toxic solvents could contribute to the creation of eco-friendly industrial processes. The next major step is the scale-up of the immobilization process for commercial applications. This involves improving enzyme loading consistency, optimizing reactor design, and carrying out cost–benefit analyses to ensure that the technology is commercially competitive with existing alternatives.

## Figures and Tables

**Table 1:** Immobilization of PCrL with different organic and hybrid supports. Immobilization of PCrL with different organic and hybrid supports.

Methods	PCrL-Support	Initial Enzyme Activity ^1^ (U/g of Ball)	Unbound Enzyme Activity (U/g of Ball)	Bound Enzyme Activity (U/g of Ball)	Immobilization Yield (%)	Activity Recovered (%)
Encapsulation	SA (2%)	40	2.35	7.5	94	19.9
	SA (2%)-CS (0.8%)	45	1.85	11.7	95	27.2
Encapsulation–Adsorption	SA (2%)-ZE (1.2%)	37	0	17.5	100	47.3

^1^ The lipase activity was measured at pH value 9 using TC8 as a substrate.

**Table 2:** Comparison of the kinetic and thermodynamic parameters of the free and immobilized PCrL (on SA and CaCO_3_ beads).

Enzyme	Temperature (K)	t_1/2_ (h)	*k*_d_ (h^−1^)	D (h)	ΔH_d_ (kJ·mol^−1^)	ΔG_d_ (J·mol^−1^)	ΔS (J·mol^−1^ K^−1^)	E_d_ (kJ·mol^−1^)	E_a_ (kJ·mol^−1^)
Free PCrL	313	1	0.346	6.656	−16.80	2	−53.68	−7.28	−0.60
	323	0.5	0.693	3.323	−17.10	−1.12	−52.93		
	333	0.25	0.326	7.064	−17.41	−1.16	−52.27		
PCrL-SA	313	4	0.173	13.312	−13.16	−1.08	−42.04	−3.64	−0.03
	323	3	0.231	10.013	−13.46	2.85	−41.68		
	333	2	0.346	6.656	−13.77	1.16	−41.35		
PCrL-CaCO_3_	313	6	0.115	13.312	−13.18	−1.15	−42.10	−3.66	−0.033
	323	4	0.173	13.312	−13.48	−1.16	−41.73		
	333	3	ar0.231	10.013	−13.77	−1.16	−41.34		

## Data Availability

The original contributions presented in this study are included in the article. Further inquiries can be directed to the corresponding author.
